# Supplementary material for: Connecting high-resolution 3D chromatin organization with epigenomics
Source: Nat Commun. 2022 Apr 19;13:2054. doi: 10.1038/s41467-022-29695-6 (PMC9018831; doi:10.1038/s41467-022-29695-6)
Supplement: Supplementary file 1 — Supplementary Information [file 41467_2022_29695_MOESM1_ESM.pdf]

Supplementary materials for paper titled "Connecting high-resolution 3D chromatin organization with epigenomics"

Fan Feng[1], Yuan Yao[2], Xue Qing David Wang[3], Xiaotian Zhang[4], Jie Liu[1*]

[1] Department of Computational Medicine & Bioinformatics, University of Michigan, Ann Arbor, MI, USA
[2] Department of Computer Science & Engineering, University of Michigan, Ann Arbor, MI, USA
[3] Division of Hematology, Department of Medicine, Keck School of Medicine, University of Southern California, Los Angeles, CA, USA
[4] Department of Pathology, University of Michigan, Ann Arbor, MI, USA
contact: drjieliu@umich.edu

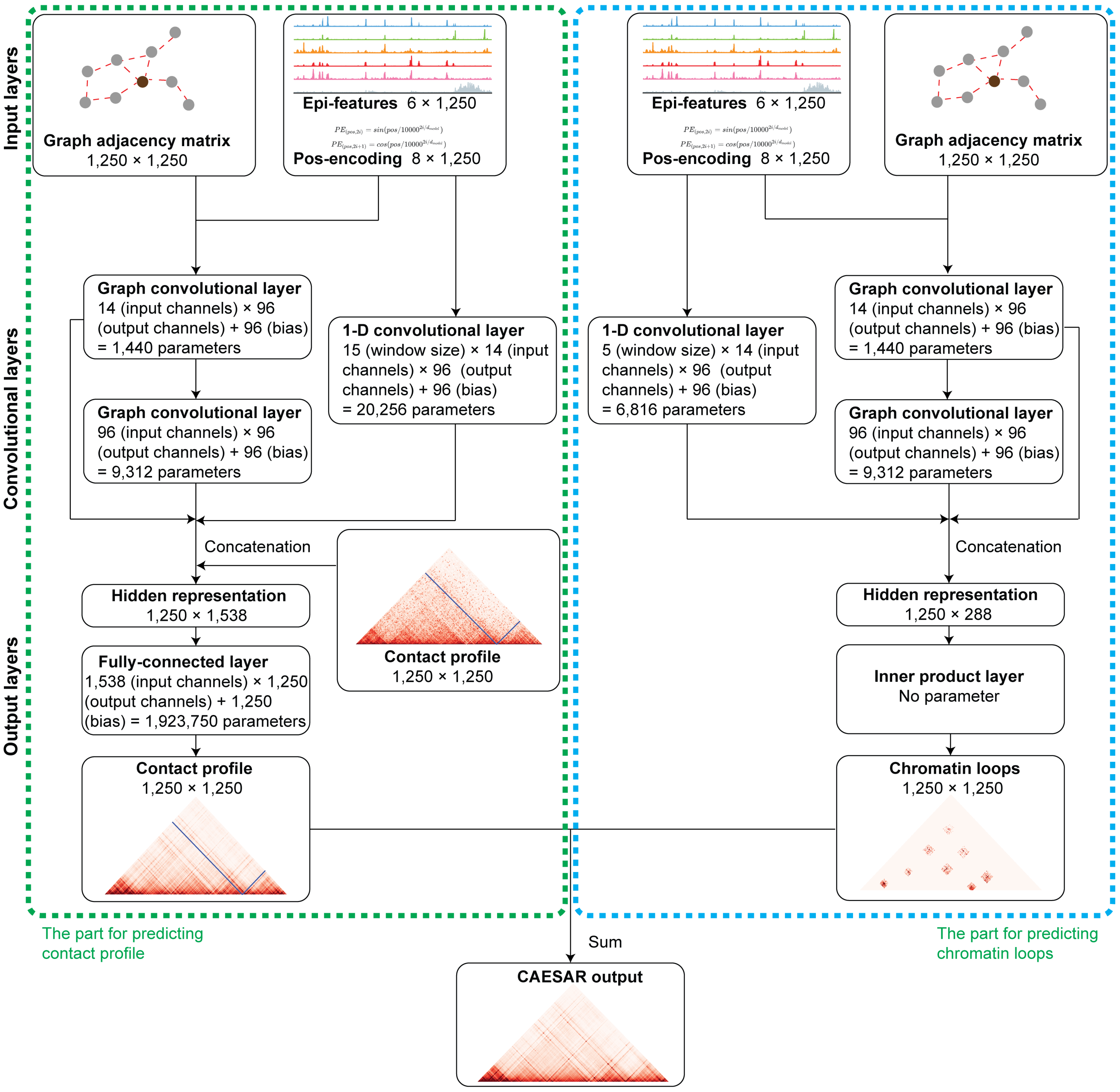

Supplementary Figure 1: Model structure details.

The model includes two parts — one for predicting chromatin loops and the other for predicting the contact profile, and each part includes input layers, convolutional layers, and output layers. At last, the outputs of the two parts are summed up to generate the final output.

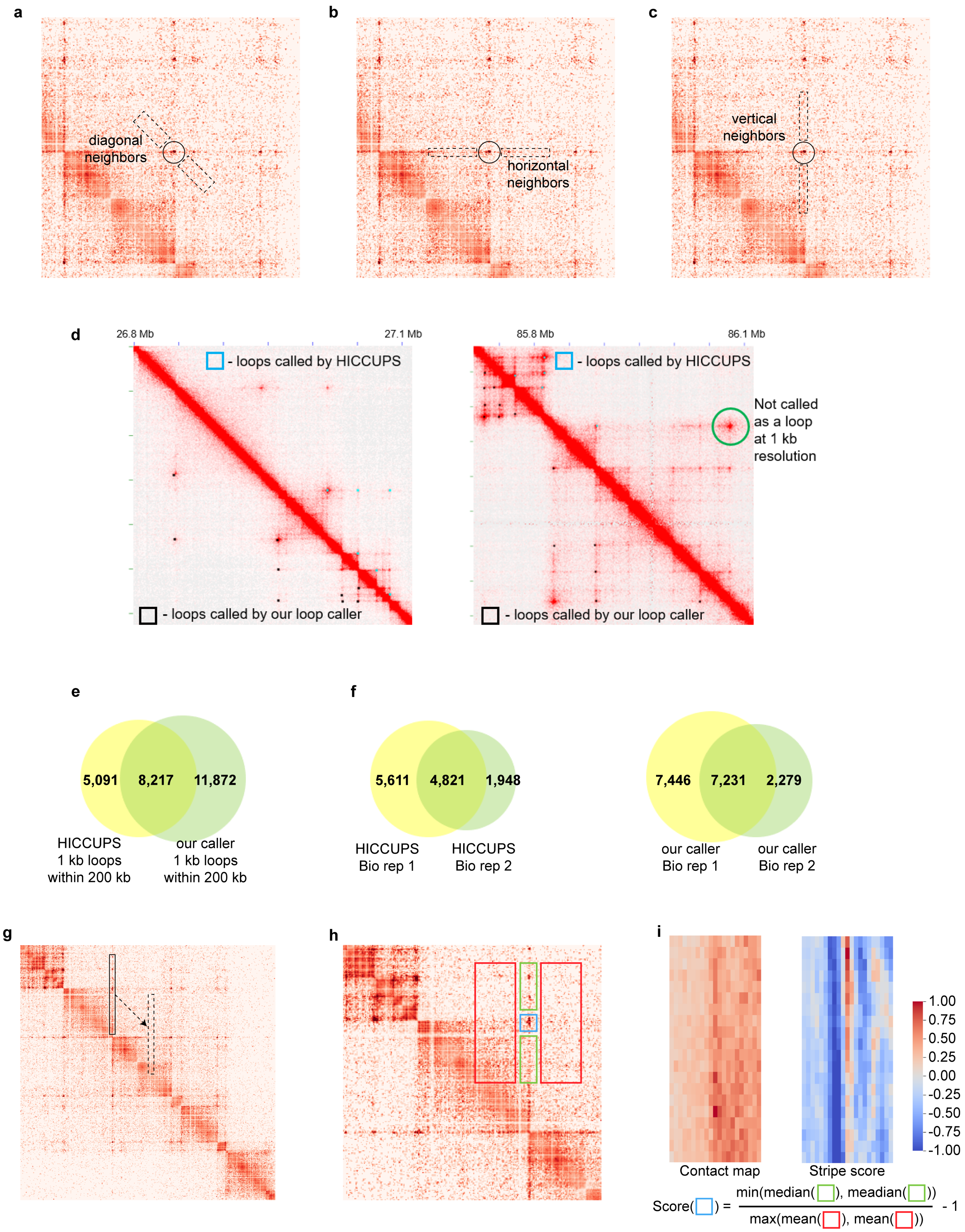

Supplementary Figure 2: The illustration of the loop caller (a-f) and stripe caller(g-i) in our study.
a, b, c, Three neighboring regions are used to calculate the expectation of a center pixel. **d**, The comparison of HICCUPS and our loop caller's results in two example regions. **e**, The Venn diagram compares HICCUPS and our loop caller's results in the HFF Micro-C contact map. **f**, The overlap ratio between reported loops on two replicates of HFF Micro-C is similar between HICCUPS and our loop caller. **g**, Step 1: A narrow and long sliding window moves along the diagonal to identify candidate vertical stripes. **h**, Step 2: For each pixel on the candidate stripe, five windows are selected and a "stripe score" is calculated for evaluating whether it is on a stripe. **i**, An example of original contacts versus stripe scores illustrates that positive scores indicate potential stripes.

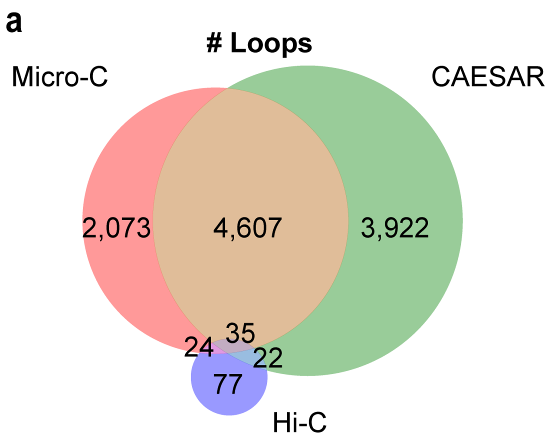

Supplementary Figure 3: CAESAR trained with surrogate Hi-C contact maps still accurately predicts Micro-C loops and stripes.

a, The Venn diagram of the loops called from 1) the input Hi-C contact map, 2) the CAESAR-imputed contact map (trained with surrogate Hi-C), and 3) the observed Micro-C contact map. **b**, The Venn diagram of the stripes called from 1) the input Hi-C contact map, 2) the CAESAR-imputed contact map (trained with surrogate Hi-C), and 3) the observed Micro-C contact map.

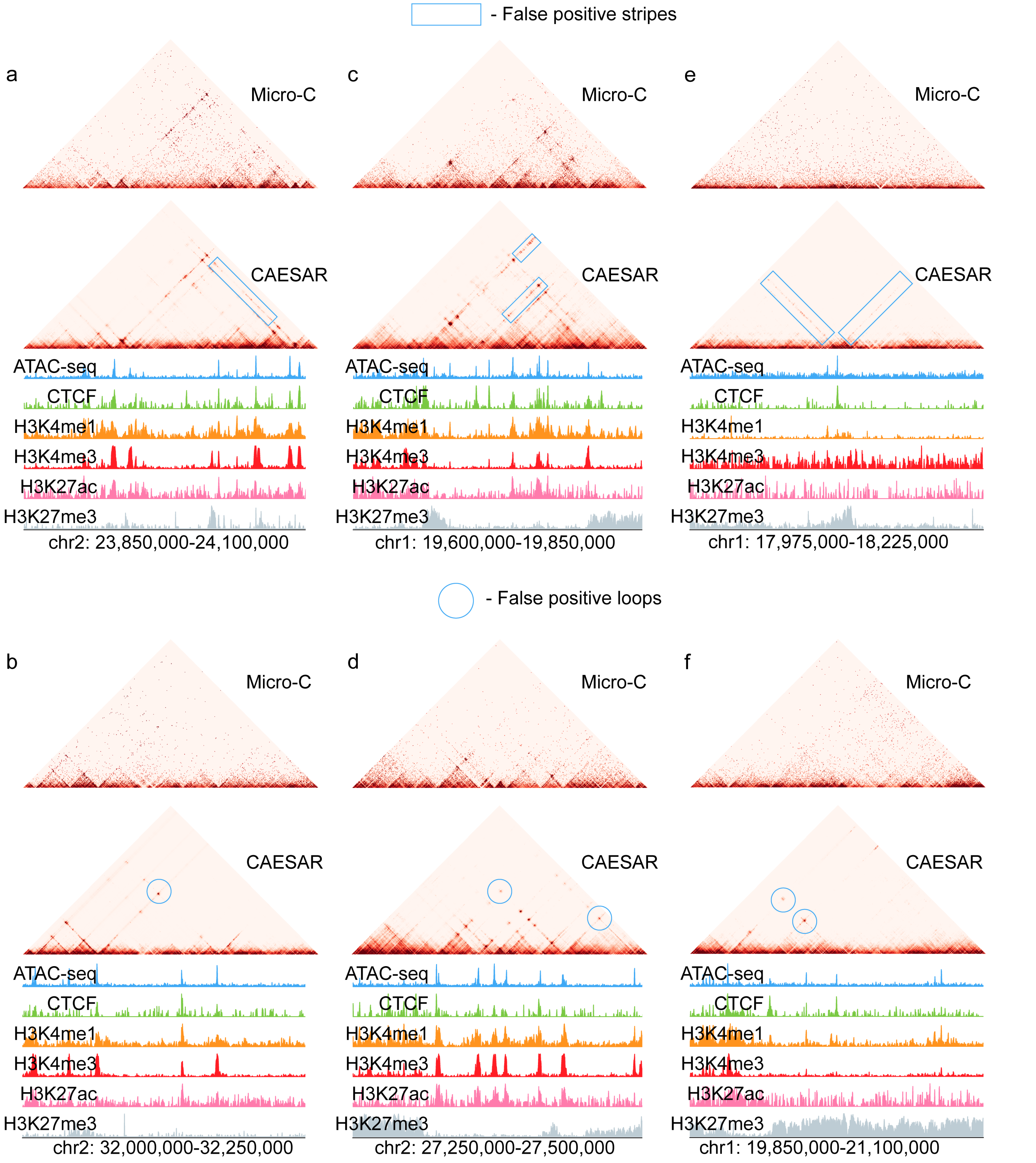

Supplementary Figure 4: The analysis of CAESAR's false-positive stripes and loops.
a, b, Due to the limited sequencing depth of Micro-C, some patterns are stripe-like or loop-like but not enriched enough for the callers to recognize. CAESAR enhances some of these structures to generate a false-positive but much clearer stripe or loop. **c, d**, When there are a set of CTCF/ATAC-seq peaks in a small region without clear TAD separation, CAESAR may generate false-positive stripes on the peaks or loops between the peaks. **e, f**, "Isolated" CTCF and ATAC-seq peaks in repressed regions may result in false-positive stripes and loops.

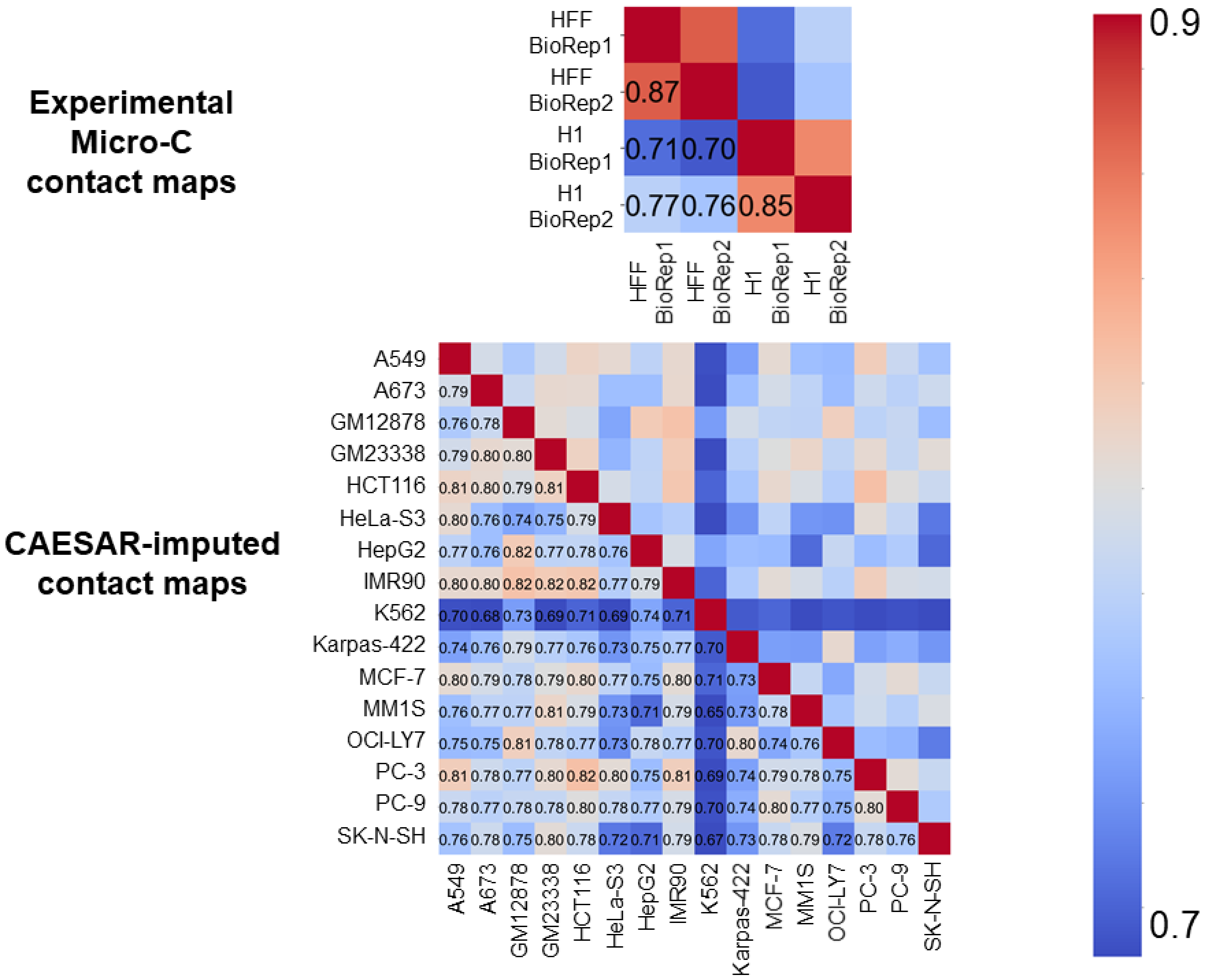

Supplementary Figure 5: The heatmap (above) of HiCRep reproducibility scores between Micro-C contact maps from HFF/H1 biological replicates and the heatmap (below) of HiCRep reproducibility scores between the imputed contact maps of the sixteen cell lines used in our study.

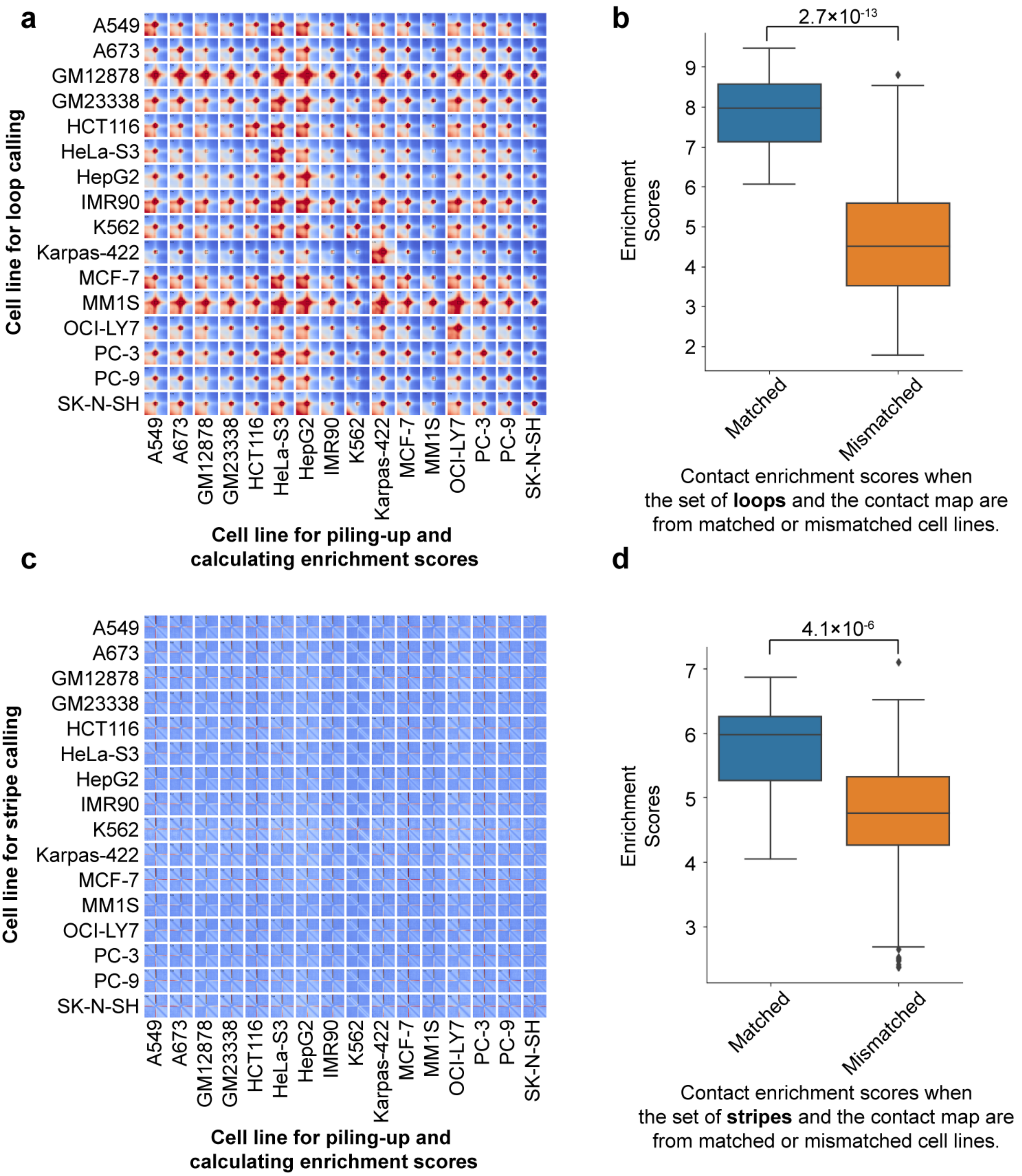

Supplementary Figure 6: CAESAR predicts cell-type variability of fine-scale structures. (**a** and **c**). Sixteen sets of loops and stripes were called from CAESAR-imputed contact maps of sixteen cell lines, and APA analysis of these loop/stripe regions was carried out across sixteen cell lines, (**b** and **d**). Contacts are significantly more enriched when the set of loops/stripes and the contact map are from the matched cell line. (One-sided t-test with n[Matched pairs]=16 and n[Mismatched pairs]=120. In the boxplots, the center line indicates median; the box limits are upper and lower quartiles; the whiskers are 1.5×interquartile range; the points are outliers.)

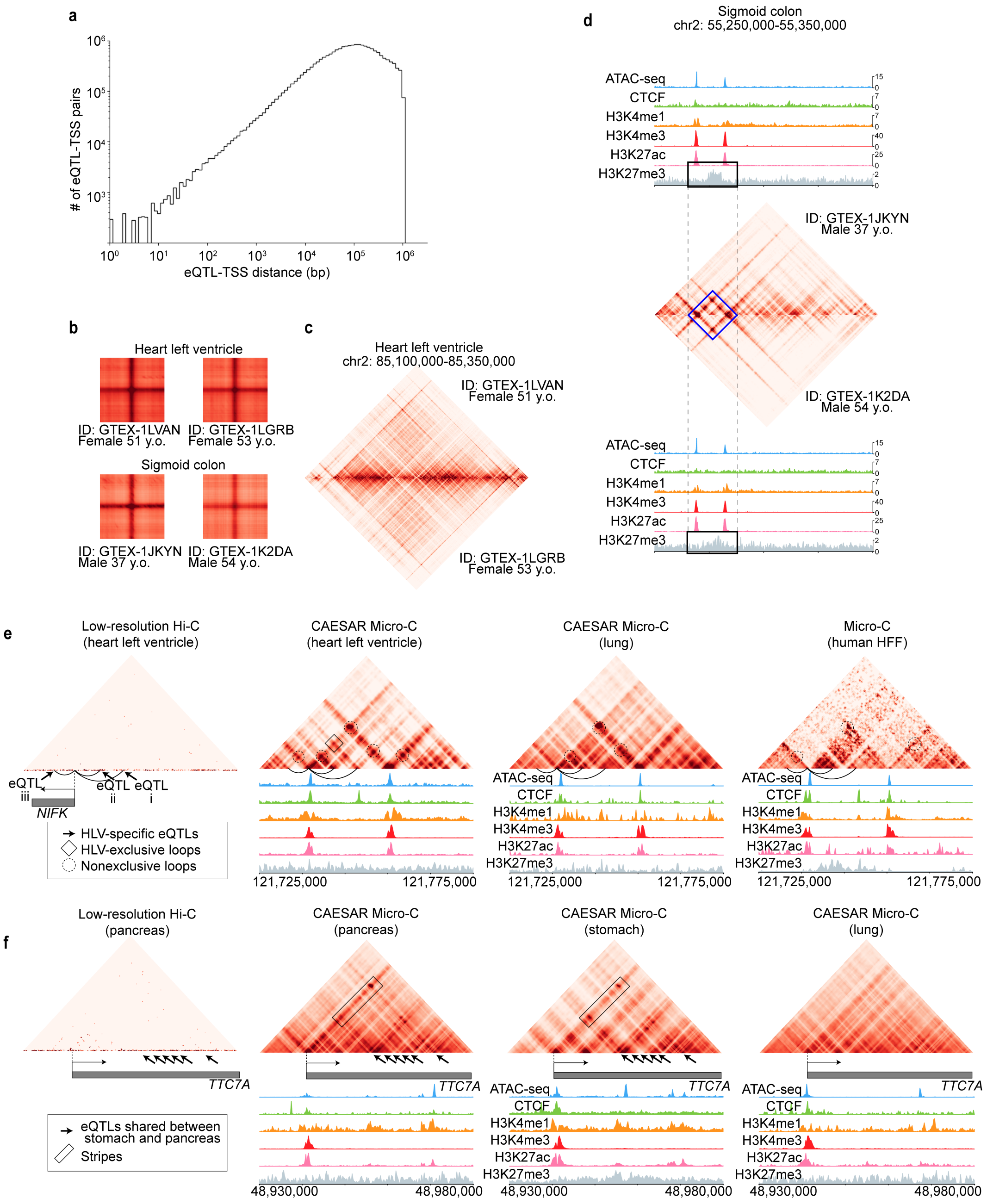

Supplementary Figure 7: The imputation of high-resolution contact maps and eQTL-TSS enrichment analysis for human tissues.

a, The distribution of eQTL-TSS distances in the 12 human tissues and cell lines demonstrates that about 50% of eQTL-TSS pairs are less than 100 kb apart, which are hard to identify on low-resolution Hi-C contact maps. **b**, The eQTL-TSS pile-up results for different donors from the heart left ventricle and sigmoid colon are consistent. **c**, The example region from the imputed contact maps of two heart left ventricle donors illustrates that, besides eQTL pile-up results, the imputed tissue contact maps are mostly consistent between individuals. **d**, A counter-example — a loop is observed on the imputed contact map of sigmoid colon from donor GTEX-1JKYN but not donor GTEX-1K2DA, which is related to a more clear H3K27me3 peak in donor GTEX-1JKYN's epigenomic features. **e**, The loops between gene *NIFK*'s TSS and its three eQTLs specific in heart left ventricle (HLV), which cannot be observed on the low-resolution Hi-C contact map, appear on the CAESAR-imputed contact map of HLV. Although all three eQTLs are HLV-specific, only the loop between *NIFK* TSS and eQTL i is HLV-exclusive; while the other two loops can also be observed on the CAESAR-imputed contact map of lung and the Micro-C contact map of HFF, respectively. **f**, A series of gene *TTC7A*'s eQTLs are shared by stomach and pancreas, and both loops and stripes are observed on the CAESAR-imputed contact maps of the two tissues. As a reference, the contacts are not observed on the low-resolution Hi-C contact map of pancreas and less enriched on the CAESAR-imputed contact maps of lung.

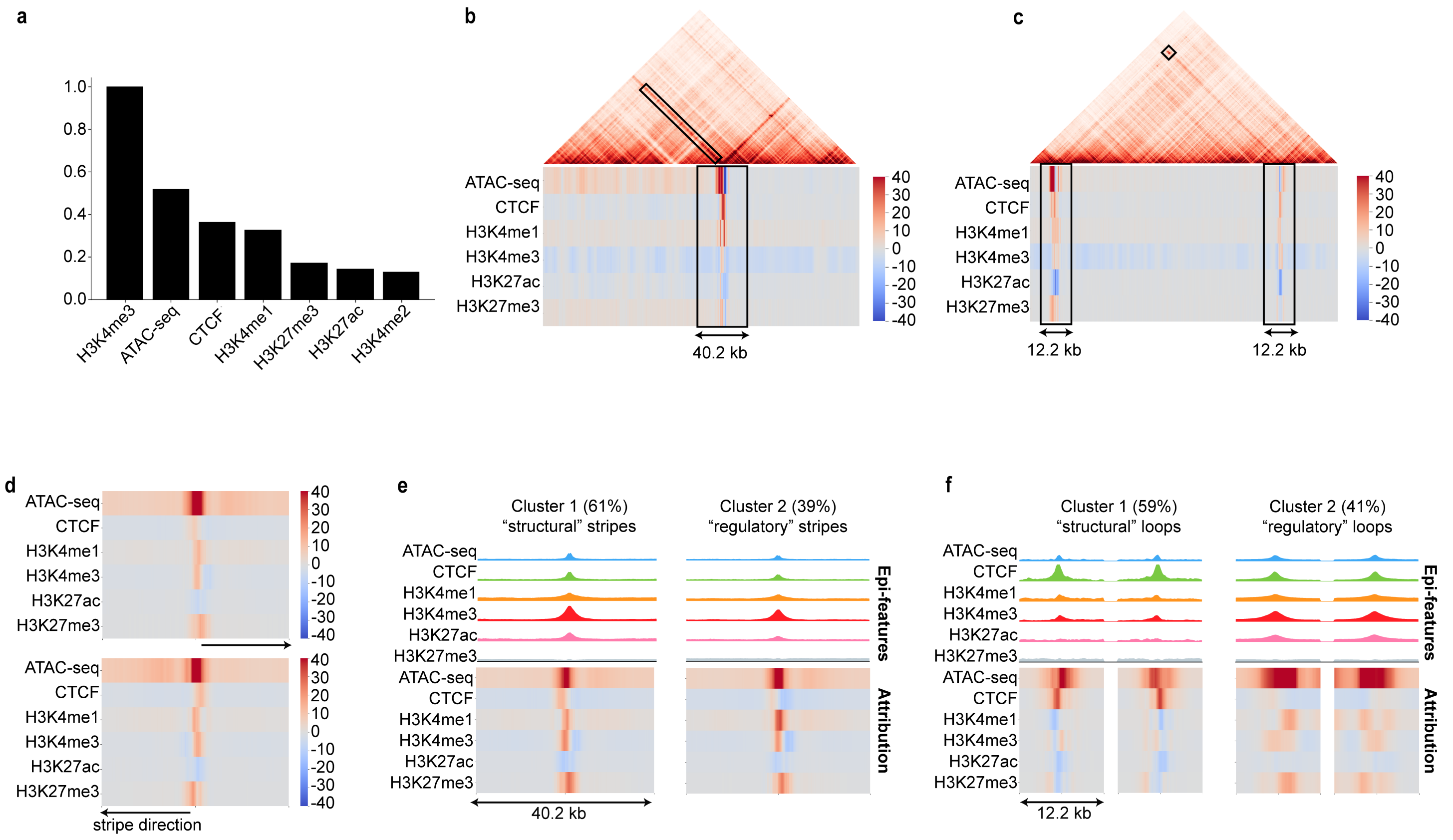

Supplementary Figure 8: Attributing CAESAR's outputs towards input epigenomic features.
a, By attributing the entire contact map to the 7 epigenomic features, we obtained the overall attribution for each epigenomic features. Since H3K4me2 is less commonly profiled and also contributes less, we can leave it out from the 6-epi model. **b**, The attribution is calculated at a stripe region. In the genome-wide attribution analysis of stripes, we collected attribution from the 40.2 kb region centered at the anchor of each stripe. **c**, The attribution is calculated at a loop region. In the genome-wide attribution analysis of loops, we collected attribution from the 12.2 kb regions centered at the anchors of each loop. **d**, The average attribution of stripes spanning to downstream and upstream directions repsectively. **e**, The clustering and embedding of all stripes' attribution illustrate that there are two clusters of stripes, which means the model has learned two major patterns indicating stripes. The average epigenomic features and attribution for each cluster are visualized. **f**, The clustering and embedding of all loops' attribution illustrate that there are two clusters of loop, which means the model has learned two major patterns indicating stripes. The average epigenomic features and attribution for each cluster are visualized.

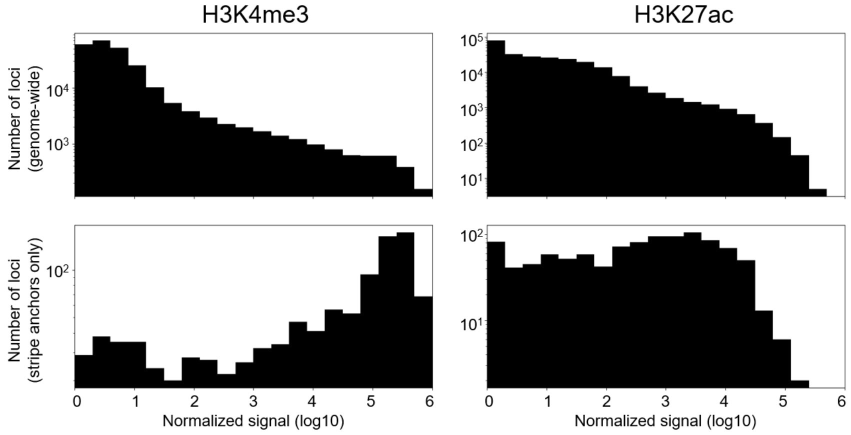

Supplementary Figure 9: The histograms of H3K4me3 and H3K27ac signal distribution in the genome *v.s* at stripe anchors. It is observed that most stripe anchors are highly enriched for H3K4me3. For the 1,000 loci with the highest H3K4me3 signal, 374 of them are stripe anchors; for the 1,000 loci with the highest H3K27ac signal, only 50 of them are stripe anchors. Therefore, although H3K4me3 and H3K27ac are both enriched in active regions, H3K4me3 shows a much higher enrichment at stripe anchors, and therefore CAESAR connects stripes to positive H3K4me3 attribution. Instead, CAESAR is likely to regard H3K27ac as a feature related to "active regions but not stripes" and gives negative attribution.

Supplementary Table 1. CAESAR-imputed tissues and cell lines

**Tissue**		
Adrenal gland	Ascending aorta	Body of pancreas
Breast epithelium	Esophagus muscularis mucosa	Esophagus squamous epithelium
Gastrocnemius medialis	Gastroesophageal sphincter	Heart left ventricle
Lung	Ovary	Pancreas
Peyer's patch	Prostate gland	Right atrium auricular region
Sigmoid colon	Spleen	Stomach
Suprapubic skin	Testis	Thoracic aorta
Thyroid gland	Tibial artery	Tibial nerve
Transverse colon	Upper lobe of left lung	Uterus
Vagina		
**Cell line**		
A549	A673	GM12878
GM23338	HCT116	HeLa-S3
HepG2	IMR-90	K562
Karpas-422	MCF-7	MM1S
OCI-LY7	PC-3	PC-9
SK-N-SH		
**Primary cell**		
B cell	CD14-positive monocyte	Astrocyte
Endothelial cell of umbilical vein	Fibroblast of dermis	Fibroblast of lung
Foreskin fibroblast	Foreskin keratinocyte	Keratinocyte
Mammary epithelial cell	Osteoblast	Skeletal muscle myoblast
***In vitro* differentiated cell**		
Bipolar neuron	Cardiac muscle cell	Hepatocyte
Myotube	Neural progenitor cell	Smooth muscle cell

Supplementary Table 2. Data sources of Hi-C/Micro-C contact maps (with link)

Contact map	Cell line	4DN/GEO Accession
Micro-C	H1-hESC	4DNES21D8SP8
	HFF	4DNESWST3UBH
	mouse ESC	4DNES14CNC1I
Hi-C	H1-hESC	4DNES2M5JIGV
	HFF	4DNES2R6PUEK
	mouse ESC	4DNESKKSKG7Y
	IMR-90	4DNES1ZEJNRU
	K562	4DNESI7DEJTM
	GM12878	4DNES3JX38V5
	Pancreas	GSE87112
	Lung	GSE87112
	Heart left ventricle	GSE87112

Supplementary Table 3. Data sources of epigenomic tracks for training

H1-hESC	
ATAC-seq	https://www.ncbi.nlm.nih.gov/geo/query/acc.cgi?acc=GSM2386576
Rad21	https://www.encodeproject.org/experiments/ENCSR000ECE/
CTCF	https://www.encodeproject.org/experiments/ENCSR000BNH/
Nanog	https://www.encodeproject.org/experiments/ENCSR000BMT/
H3K4me1	https://www.encodeproject.org/experiments/ENCSR271TFS/
H3K4me2	https://www.encodeproject.org/experiments/ENCSR322MEI/
H3K4me3	https://www.encodeproject.org/experiments/ENCSR443YAS/
H3K9ac	https://www.encodeproject.org/experiments/ENCSR441UHO/
H3K9me3	https://www.encodeproject.org/experiments/ENCSR883AQJ/
H3K27ac	https://www.encodeproject.org/experiments/ENCSR880SUY/
H3K27me3	https://www.encodeproject.org/experiments/ENCSR928HYM/
H3K36me3	https://www.encodeproject.org/experiments/ENCSR476KTK/
H3K79me2	https://www.encodeproject.org/experiments/ENCSR301HRV/
HFF	
DNase-seq	https://www.encodeproject.org/experiments/ENCSR672EWY/
CTCF	https://data.4dnucleome.org/experiment-set-replicates/4DNESJGIALEC/
H3K4me1	https://data.4dnucleome.org/experiment-set-replicates/4DNESR96HCCM/
H3K4me2	https://data.4dnucleome.org/experiment-set-replicates/4DNESWK53WP1/
H3K4me3	https://data.4dnucleome.org/experiment-set-replicates/4DNES8ZSCTFJ/
H3K27ac	https://data.4dnucleome.org/experiment-set-replicates/4DNESV6NQ665/
H3K27me3	https://data.4dnucleome.org/experiment-set-replicates/4DNESNNHF597/

Supplementary Table 4a. Data sources of epigenomic tracks for human tissues (with link)

Tissue	Donor*	ATAC-seq**	CTCF	H3K4me1	H3K4me3	H3K27ac	H3K27me3
Ascending aorta	F 51	422IIZ***	846JKO	202XTW	645FBM	982QIF	103QHX
	F 53	968TPO	555DCD	707AEW	122LOZ	069UMW	589GII
Body of pancreas	M 37	152PSA	572DUJ	827NKO	876DCP	520BIM	977CEC
	M 54	464TKV	687APM	348TQM	554RQQ	596PFU	774CFO
Breast epithelium	F 51	846ZBX	661NXJ	263XKR	568QQU	081OTO	134LLK
	F 53	654UYP	304XUZ	553IAW	416AUW	034ZKE	770WSE
Esophagus muscularis mucosa	F 51	686ZKE	443WKD	701GIE	403PEI	894MOX	200AFX
	M 54	609GST	073TPC	674WSL	077HGR	705BTW	543UBL
Esophagus squamous epithelium	F 51	096BPX	266UTR	658EVN	773PIU	204TAU	049FUB
	F 53	579BNV	756URL	121RSS	508UPW	909UAG	057BFO
	M 37	944JCE	559KAB	525JJM	621MTP	522MTS	188HXK
Gastrocnemius medialis	F 51	823ZCR	355ALW	499VCQ	098OLN	601VHO	453MSI
	F 53	689SDA	428BKN	776EAH	785DJD	736ALU	201OSX
	M 37	258JCL	594NSU	148FWR	206STN	801IPH	519WQH
	M 54	308HPZ	998NQG	161HZJ	972ETR	948YYZ	423LXQ
Heart left ventricle	F 51	117PYB	718SDR	449FRQ	181ATL	702OVJ	613PPL
	F 53	851EBF	544APK	438QZN	901SIL	854OXF	988JLN
Peyer’s patch	F 51	261RWJ	542SCB	874HIG	684EPX	249IKQ	491FDG
	F 53	689DSM	375VXU	621BZD	878KIY	837SGJ	982PLJ
	M 37	954AJK	419ANE	416ZMW	349GPJ	440PMP	632SLJ
	M 54	455GUW	568IVD	912XAL	998QKF	758KRK	735VKO
Prostate gland	M 37	564FZH	946MNG	155XVP	153NDQ	841AJO	690CSD
Right atrium auricular region	F 51	278SKG	232OFD	817FGU	954TSY	668EVA	459CKR
	F 53	984SQJ	401KRN	368ORV	791KFQ	593KDJ	793PLF
Sigmoid colon	M 37	548QCP	721AHD	181HTE	960AAL	807XUB	734ZTQ
	M 54	086OGH	857RJQ	775LGE	172LVU	937EVN	860GPM
Spleen	F 51	078EBD	595BPR	831EDZ	589DBF	668GBL	161FEJ
	F 53	128GBN	601FEB	659RJP	197QDK	726HTS	826MTK
	M 54	850YHJ	225YGX	635IRN	377ILM	593INW	080JPX
Stomach	F 51	641ZPF	361KVZ	009RJD	492BHN	751BHO	330MAM
	F 53	006IMH	185CCV	903QBX	489ZLL	133NBJ	357ROS
	M 37	177NIJ	618QYE	493MQY	843UEZ	944KAZ	227DGG
Testis	M 37	866ODX	753RME	956VQB	611DJQ	136ZQZ	503QSX
Thyroid gland	F 51	450PWF	955BIB	497OVD	309UVT	500YBS	586DVD
	M 37	749MUH	505ZGX	906YES	901BRV	597BWL	748LUA
	M 54	549NRK	033KMZ	639NMN	975NOU	203KCB	582PKH
Tibial artery	M 37	102RSU	079YAP	960VRR	780CNW	891BTJ	764OHK
Tibial nerve	F 51	401ESD	793YAD	338PGG	677MOE	778QHG	992XOO
	F 53	100TUY	875NEW	850RVA	314SPW	771YJT	611YUJ
	M 37	484UAU	434XLP	981CTV	384MUF	516LQO	860ZCZ
	M 54	508FVM	689VEF	590NNJ	464TRM	091KXI	662ASZ
Transverse colon	F 51	386HAZ	449SEF	500QVK	315EZG	792VLP	604QMH
	F 53	404LLJ	236YGF	791LZY	933BVL	208QRN	840VWD
	M 37	668VCT	608WPS	516QFO	813ZEY	640XRV	643KID

Upper lobe of left lung	F 51	323UTX	799TJD	238WIK	429VWL	453MUW	706OFD
	F 53	702DPD	224WWI	263OXW	208WDY	738SXD	859MXQ
	M 37	164WOF	027FSZ	595MTV	074WIB	505YFA	469YCE
	M 54	650FLQ	463XCZ	348FGT	701FGA	948TOS	050LBS
Uterus	F 53	129BZE	684PGO	035ONO	354ZUG	249INE	111DTF
Vagina	F 51	733YNW	655ECZ	495RJG	647HAQ	346FVK	278TQE
Adrenal gland	mixed	277KRY	899JSO	455JUO	620TXL	094VJC	181JFC
Gastroesophageal sphincter	mixed	260ZIV	298ZPF	134KZX	037GFN	600TOW	965BLU
Lung	mixed	647AOY	000DMH	356ANC	466DZW	540ADS	204NFO
Ovary	mixed	712PYJ	548DDS	113AFY	139TLA	268JQE	037SNV
Pancreas	mixed	595HZQ	000DND	984UHU	315LPR	402HFW	486NDF
Suprapubic skin	mixed	709IYR	485VQV	374XIN	362QYU	413QLR	410BWN
Thoracic aorta	mixed	344ZTM	549TXG	803IBD	930HLX	318HUC	939RLS

∗ In this column, "F" and "M" indicate female and male, and numbers indicate the donors' age. "Mixed" indicates the datasets are from multiple donors.
∗∗ For tissues or cell lines without available ATAC-seq data, we collected DNase-seq instead.
∗ ∗ ∗ "422IIZ" is short for "ENCSR422IIZ". In this and the following tables, "ENCSR" is omitted for all accessions.

Supplementary Table 4b. Data sources of epigenomic tracks for human cell lines (with link)

Cell line	DNase-seq	CTCF	H3K4me1	H3K4me3	H3K27ac	H3K27me3
A549	000ELW	000DNA	636PIN	000DPD	778NQS	000AUJ
A673	346JWH	611JJS	521IZK	435FGK	714TJD	747BYL
GM12878	000EJD	000DZN	000AKF	057BWO	000AKC	000DRX
GM23338	004SUL	987GXT	249YGG	657DYL	729ENO	386RIJ
HCT116	000ENM	240PRQ	161MXP	333OPW	661KMA	810BDB
HeLa-S3	959ZXU	000DLO	000APW	340WQU	000AOC	000APB
HepG2	149XIL	000DUG	000APV	575RRX	000AMO	000AOL
IMR-90*	477RTP	000EFI	831JSP	087PFU	002YRE	431UUY
K562	000EKS	000DWE	000EWC	668LDD	000AKP	000EWB
Karpas-422	019JDO	113REG	306VSH	910XKX	660IQS	963HAR
MCF-7	000EPJ	560BUE	493NBY	985MIB	752UOD	761DLU
MM1S	458LIB	402IDP	094VCE	361FWQ	758OEC	404LJZ
OCI-LY7	489NAM	027HML	060WGK	005SXO	447ZGY	752KQT
PC-3	052AWE	359LOD	566UMF	275NCH	826UTD	881TWJ
PC-9	940NLN	243INX	913MGR	441JWF	769FOC	726LZG
SK-N-SH	000EPZ	541AMF	661BMA	975GZA	564IGJ	914QOK

∗ IMR-90 is not a cancer cell line.

Supplementary Table 4c. Data sources of epigenomic tracks for primary cells (with link)

Primary cell	DNase-seq	CTCF	H3K4me1	H3K4me3	H3K27ac	H3K27me3
B cell	381PXW	000AUV	290YLQ	000DQR	000AUP	162DGX
CD14-positive monocyte	000EPK	000ATN	000ASM	000DWL	000ASJ	000DWM
Astrocyte	000EPM	000AOO	000AOT	000AOU	000AOQ	000AOR
Endothelial cell of umbilical vein	000EOQ	000ALA	000AKL	578QSO	000ALB	000AKK
Fibroblast of dermis	000EPO	000APM	000ARV	000APR	000APN	000APO
Fibroblast of lung	000EPR	000DWY	000AMU	915QOL	000AMR	000AMS
Foreskin fibroblast	153LHP	000DUH	367HVD	813CFB	917QEH	417IEJ

Foreskin keratinocyte	035RVH	817HTJ	027BAJ	075OQB	666TFS	377MRR
Keratinocyte	000ELH	000DNC	000ALI	970FPM	000ALK	000DWU
Mammary epithelial cell	000ENV	000DUS	521FND	000DUQ	000ALW	000ALX
Osteoblast	000ELJ	000APF	000APJ	000ATH	000APH	000AQS
Skeletal muscle myoblast	000EOO	000ANE	000ANI	596NOF	000ANF	000ANG

Supplementary Table 4d. Data sources of epigenomic tracks for *in vitro* differentiated cell (with link)

In vitro differentiated cell	DNase-seq	CTCF	H3K4me1	H3K4me3	H3K27ac	H3K27me3
Bipolar neuron	626RVD	619IUE	301AEA	849YFO	905TYC	472SEY
Cardiac muscle cell	842KCP	713SXF	276OLB	652QNW	000NPF	864LRY
Hepatocyte	364MFN	252QYR	689QUB	442ZOI	507UDH	637RLN
Myotube	000EOP	000ANS	000ANX	000ANZ	000ANV	000ATI
Neural progenitor cell	963ALV	125NBL	274OIJ	661MUS	449AXO	139PIA
Smooth muscle cell	248CME	261VAS	130IMV	515PKY	210ZPC	143RMH

Supplementary Table 5. The numbers of original and tissue/cell type-specific eQTLs

Tissue/Cell line	Original eQTLs	Filtered specific eQTLs
Adrenal gland	691,864	31,538
GM12878	1,942,811	325,793
Heart left ventricle	1,005,665	68,585
IMR-90	338,487	14,870
Lung	1,664,707	128,509
Pancreas	962,413	74,547
Sigmoid colon	1,038,961	40,011
Spleen	927,548	56,628
Stomach	834,210	23,732
Testis	2037163	635,726
Tibial nerve	2,352,070	343,799
Transverse colon	1,190,047	53,334

Supplementary Note 1   Data collection and processing

The datasets used in our cross-validation experiments include Hi-C contact maps, epigenomic features, and Micro-C contact maps for three cell lines — hESC, mESC and HFF. Micro-C and Hi-C contact maps of HFF, hESC, and mESC were downloaded from the 4DN data portal [1]. Chromatin accessibility (ATAC-seq and DNase-seq) data of HFF, hESC, and mESC were downloaded from ENCODE database [2]. Twelve ChIP-seq signals of mESC and hESC (CTCF, Rad21, Nanog, H3K4me1, H3K4me2, H3K4me3, H3K9ac, H3K9me3, H3K27ac, H3K27me3, H3K36me3, and H3K79me2) were downloaded from ENCODE database and CistromeDB [3, 4]. Due to the unavailability of HFF ChIP-seq data, we used CUT&RUN as an alternative, and six HFF CUT&RUN signals (CTCF, H3K4me1, H3K4me2, H3K4me3, H3K27ac, and H3K27me3) were downloaded from the 4DN data portal[1].

For imputing high-resolution contact maps of additional human tissue types and cell lines, we collected Hi-C contact maps and epigenomic signals of human tissues and cell lines. Hi-C contact maps with more than 1 billion contacts (K562, IMR-90, and GM12878) were downloaded from the 4DN data portal. The epigenomic signals from 91 samples were downloaded from ENCODE (Supplementary Note 2).

To validate CAESAR's performance in predicting the interactions between regulatory elements, we collected eQTLs and CRISPRi data. The eQTL data of 10 human tissues (adrenal gland, sigmoid colon, transverse colon, heart left ventricle, lung, tibial nerve, ovary, pancreas, spleen, and stomach) and 2 human cell lines (GM12878 and IMR-90) were downloaded from GTEx Analysis Release V8 [5]. The K562 CRISPRi data were downloaded from the original study of Fulco *et. al.* [6].

To evaluate CAESAR's performance in different genomic regions, we collected phastCons scores and repli-seq data to separate all regions into different groups. The 100-way phastCons scores of hg38 were downloaded from UCSC genome browser [7], and the repli-seq data were downloaded from the 4DN data portal.

The detailed metadata is summarized in Supplementary Tables 2 and 3. All Hi-C contact maps were processed into 1 kb resolution and then linearly interpolated to 200 bp resolution; all Micro-C contact maps and epigenomic signals were processed into 200 bp resolution. All Micro-C contact maps were OE-normalized (i.e., observed/expected normalized for each stratum). All mouse data in our analysis used mm10 reference genome, and all human data in our analysis used hg38 reference genome.

Supplementary Note 2   Collecting epigenomic signals from ENCODE database

We searched the ENCODE Data Matrix (https://www.encodeproject.org/matrix/?type=Experiment) to collect epigenomic signals for imputing high-resolution contact maps. We limited the organism to *homo sapiens*, and identified all biosamples (tissues, cell lines, primary cells, and *in vitro* differentiated cells) with all of the following signals — ATAC-seq/DNase-seq, CTCF, H3K4me1, H3K4me3, H3K27ac, and H3K27me3. For a specific human tissue, there are two outcomes. If all six epigenomic tracks are available for individual donors, then we will impute the contact map for these individual donors separately. If we do not have sufficient epigenomic tracks for imputing for the individual donors, then we will only impute one contact map for the specific human tissue (referred to as "mixed-donor tissue"). In the end, we identified 91 sets of epigenomic signals from 50 individual donors for 21 tissue types, 7 mixed-donor tissues, 16 cell lines, 12 primary cells, and 6 *in vitro* differentiated cells.

Supplementary Note 3   Detailed CAESAR model structures

The model includes two major parts — one for predicting chromatin loops, and the other for predicting contact profile. Each part includes consecutive input layers, convolutional layers, and output layers (Supplementary Figure 1). CAESAR captures the interpolated Hi-C contact map as a graph $\mathcal{G}$ with nodes representing genomic regions of 200 bp long, and weighted edges representing chromatin contacts. A is the adjacency matrix of $\mathcal{G}$. For both parts, the inputs include the graph adjacency matrix A and the epigenomic features X. As one 250 kb region is fed into the model each time, the dimension of the input adjacency matrix is 1250×1250. In a 6-epigenomic model, the size of the epigenomic feature matrix is 6×1250. In addition, eight positional encoding dimensions are concatenated to the epigenomic features. The positional encoding is calculated with the following method, in which pos is from 0 to 1249 and i is from 0 to 7 [8].

$$PE_{(pos,2i)} = \sin\left(pos/10000^{2i/8}\right)$$
$$PE_{(pos,2i+1)} = \cos\left(pos/10000^{2i/8}\right)$$

In deep learning models, convolutional kernels are small filters sliding through the input to extract certain patterns. When the filter is applied to an input element, it calculates the weighted sum of the element with its local neighbors. In a

convolutional layer, multiple kernels work in parallel to learn different sets of weights and extract different patterns. There are two types of convolutional layers, 1-D convolutional (Conv1D) and graph convolutional (GC) layers in CAESAR. Conv1D layers operate along the genome fiber, aggregating the epigenomic features from nearby bins. GC layers extract spatial epigenomic patterns over the spatial neighborhood specified by $\mathcal{G}$. Here, we use the GC layer

$$Y = \sigma(\tilde{A}XW)$$

in which X and Y are the input and output, $\tilde{A}$ is the normalized graph adjacency matrix, W is the trainable parameters, and σ is the $relu$ activation function [9]. GC layers provide additional structural patterns for imputing high-resolution chromatin architecture. For example, if two distant loci i and j are in the same TAD, then nodes i and j are neighbors on the graph. Therefore, when we predict the contact profile of i, the information flows from j to i in the GC layers, so that the features at j contribute to the prediction of i, and *vice versa*. The window size for each 1-D convolution kernel is 15 in the contact profile predicting part and 5 in the loop predicting part, which captures relevant features from a 3 kb and 1 kb neighborhood, respectively.

For the contact profile predicting part, the output layer is a fully-connected layer. The input of this layer is the concatenation of convolutional layers' outputs and the Hi-C contact profile, and the output is the imputed contact profile of each 200 bp bin. For the loop predicting part, the output layer is an inner product layer. This layer also takes the concatenation of convolutional layers' outputs as input, and calculates the inner product between each bin pairs' representation to predict the chromatin loops. The outputs of the two output layers are summed up to generate the final imputation result. The model includes 2 million parameters, which is much fewer than the number of elements ($\sim$15 billion) in the contact matrix.

Supplementary Note 4 Train, test and tune sets of chromosomes

We split the chromosomes into three sets of comparable sizes to train, tune, and test our CAESAR model. For hg38, the train set include chr1, 4, 7, 10, 13, 17, and 18 (total length 1,010,309,426 bp), the test set include chr2, 5, 8, 11, 14, 15, 21, and 22 (total length 1,010,520,404 bp), and the tune set include chr3, 6, 9, 12, 16, 19, 20, and X (total length 1,010,212,587 bp). For mm10, the train set include chr1, 4, 7, 8, 10, and 11 (total length 879,600,295 bp), the test set include chr2, 5, 9, 12, 14, and X (total length 874,605,583 bp), and the tune set include chr3, 6, 9, 13, 15, 16, 17, 18, and 19 (total length 879,570,794 bp).

Supplementary Note 5 Hyperparameter Tuning

CAESAR includes two hyperparameters: 1) the number of convolutional layers and 2) the number of convolutional kernels in each convolutional layer. We examine 4 different convolutional layer configurations: i) 3 GC layers, ii) 2 GC layers and 1 Conv1D layer, iii) 1 GC layer and 2 Conv1D layers, and iv) 3 Conv1D layers. In each layer, we tested 3 different numbers of convolutional kernels - 64, 96, and 128.

For each of the 12 combinations, we trained a CAESAR model with the train set and evaluated with the mean squared error (MSE) on the tune set, and the model with 2 GC layers, 1 Conv-1D layer, and 96 kernels at each layer, achieved the best performance.

Supplementary Note 6 Baselines methods and parameters

In existing literature, there are two major categories of machine learning approaches for imputing Hi-C contact maps. The first category takes low-resolution contact maps as input and treats Hi-C contact maps as 2-D images, exemplified by HiCPlus [10]. The second category predicts the contacts between every two bins with genomic or epigenomic features from the two bins, exemplified by HiC-Reg [11]. Therefore, we use HiCPlus and HiC-Reg as two baselines in our experiments.

HiCPlus is a deep-learning model with three sequential layers, in which the first and third layers are Conv2D layers, and the second layer is a fully-connected layer. Since the matrices in our study are much bigger, we accordingly increased both the number and the size of Conv2D kernels. We set the number of Conv2D kernels to be 96, and the size of Conv2D kernels to be 15$\times$15. The model was re-trained with hESC train set, in which the inputs were the Hi-C contact maps and the targets were the Micro-C contact maps.

HiC-Reg uses random forests (RF) to predict the contacts between locus i and j with the epigenomic features near i and j as well as the distance between i and j. We used a 240-tree RF to re-train the model with hESC train set, in which the combination of 6 epigenomic features were the input and the Micro-C contact maps were the targets.

Supplementary Note 7 Loop calling at 1 kb resolution

Currently published loop callers (e.g., HICCUPS and Mustache) do not directly apply to our imputed contact maps because they require a properly normalized contact map. Although HICCUPS documentation mentions the setting of "NONE" normalization, but executing the command "hiccups –cpu -k NONE" gives the error "Data not available". CAESAR currently predicts chromatin contacts within a distance range (200 kb or 1 Mb) along the diagonal, which cannot be normalized by normalization methods (including KR, VC, and VCSQRT) which require the entire contact maps. Therefore, we made a minor change to HICCUPS and implemented a new loop caller to replace our previous "fast loop calling" approach, which searches for significantly enriched pixels with respect to the neighboring regions (see details in Supplementary Note 7 in the revised manuscript). The code of our loop caller has been made available on our GitHub repository (https://github.com/liu-bioinfo-lab/caesar).

Similar to HICCUPS, it searches the contact map to identify the contact-enriched pixels. For each pixel, three neighboring regions - vertical, horizontal, and diagonal are selected (Supplementary Figure 2a-c), and the pixel's expected values are calculated as the three averages of the neighboring regions. The significance values are calculated with Poisson statistics and filtered with a Benjamini-Hochberg FDR control procedure.

To deal with sparsity in long-range interactions at 1 kb resolution, we adopted HICCUPS' λ-chunking strategy. Each pixel is assigned to a λ-chunk based on its original expected value. If $2^{(\lambda-1)/3} < Expectation < 2^{\lambda/3}$, then it is assigned to λ-chunk. All pixels with expectation below 1 (i.e., 2^0) are assigned to the 0-chunk. Then the expected values of all pixels are adjusted to $2^{\lambda/3}$ (i.e., the upper limit of their chunk). Therefore, the expectations are not too low to result in many false-positive loops from contact less-enriched regions.

We compared original HICCUPS and our loop caller (revised HICCUPS) in detecting loops from real Micro-C contact maps (Supplementary Figure 3d). For all differential loop analysis in our study, we define two loops "match" if they are less than 3 kb apart. We used "java -jar juicer_tools.jar hiccups –cpu –threads 0 -p 5 -i 10 -t 0.1 -f 0.1 -r 1000 -d 20000" to run HICCUPS. HICCUPS and our loop caller reported 13,308 loops and 20,089 loops respectively at 1 kb resolution from all chromosomes, in which 8,219 loops were called by both callers (Supplementary Figure 3e). In a control experiment in which we called loops from two biological replicates of HFF Micro-C data, HICCUPS and our loop caller showed similar overlaps (Supplementary Figure 3f). Therefore, the two callers are comparable, and our caller can also be applied to CAESAR-imputed contact maps.

Supplementary Note 8 Stripe calling with Quagga

The only paper which mentioned a stripe caller is Vian et al. [12]. However, they did not publish their tool "zebra" or provide the source code. Following their algorithm, we developed "Quagga" to call stripes on the CAESAR-imputed, Micro-C, and Hi-C contact maps. Stripes are labeled as "vertical" or "horizontal" according to their directions on the top right half of the contact map, and Quagga calls vertical and horizontal stripes separately. Quagga identifies vertical stripes as follows. First, a narrow, long sliding window anchored at the diagonal moves along the diagonal of the OE-normalized contact map. The contacts are summed up in the window at each step to obtain a 1D vector, and the peaks of the vector are identified as the candidate vertical stripes (Supplementary Figure 2g). In our work, we used a 100×1 sliding window at 1 kb resolution. However, loops, TAD boundaries, or random noise can be false positives, and therefore we further calculate a "stripe score" (Supplementary Figure 2h and 2i). For each pixel on a candidate stripe, five regions are chosen: a $i\times i$ square X centered at it, two neighboring $j\times i$ regions along the candidate stripe (upper: X_u; lower: X_w), two $(2j+i)\times j$ regions on the left (X_l) and right (X_r) and the "stripe score" is calculated as

$$Score = min(median(X_u), median(X_w))/max(mean(X_l), mean(X_r)) - 1.$$

In our work, we set i=1 and j=10. The left and right regions work as the background, and taking the maximum of the two avoids TAD boundaries to be falsely called as stripes. Calculating the median of the upper/lower regions ensures a single large value (e.g., a loop) does not increase the score. If a pixel is on a vertical stripe, then the enrichment score should be greater than 0. At last, Quagga calculates the summation of the stripe scores for each candidate stripe and output the anchor position if the summation is above a threshold. For the differential analysis between two contact maps, we say two stripes "match" if their anchor positions are less than 2 kb apart.

When we applied Quagga to the contact map imputed with 3 epigenomic features (ATAC-seq, CTCF, and H3K27ac), more than 20,000 stripes were called. Since CAESAR outputs each 200 bp bin's contact profile separately, when the input does not provide sufficient information about chromatin structures, the model outputs from neighboring bins are more random and inconsistent. The inconsistency of rows/columns may result in the calling of false-positive stripes, which explains

the over-prediction of stripes by the 3-epi model.

Supplementary Note 9 The analysis of false-positive loops and stripe

All predictive models generate false positives. Since CAESAR predicts more loops and stripes than Micro-C contact maps should be, we carefully investigated the false positives produced by CAESAR. We observed that CAESAR's false-positive loops and stripes fell into two categories.

The first category of false positives are supported by Micro-C data. Due to the limited sequencing depth of Micro-C, some patterns are stripe-like or loop-like but not enriched enough for the callers to recognize. CAESAR enhances some of these structures to generate a false-positive but much clearer stripe (Supplementary Figure 4a) or loop (Supplementary Figure 4b). By raising the FDR threshold of callers from 0.10 to 0.20, 55% of false-positive stripes and 39% of false-positive loops can be called from the real Micro-C contact map.

The second category are not supported by Micro-C data. We manually annotate these loops and stripes and found two common patterns of false positives. **1**). When there are a set of CTCF/ATAC-seq peaks in a small region without clear TAD separation, CAESAR may generate false-positive stripes on the peaks (Supplementary Figure 4c) or loops between the peaks (Supplementary Figure 4d). **2**). "Isolated" CTCF and ATAC-seq peaks in repressed regions may result in false-positive stripes (Supplementary Figure 4e) and loops (Supplementary Figure 4f). The false-positive patterns in this category may indicate these "epigenomic-3D chromatin organization" patterns frequently exist in other genomic regions and have been learned by CAESAR. For example, the second region of Figure 1b in our revised manuscript is an example region whose pattern is quite similar to Supplementary Figure 4d. Distinguishing between the two genomic regions may require additional epigenomic features or DNA sequence features.

Supplementary Note 10 Cell-type specificity of CAESAR-imputed contact maps

To show CAESAR's ability to predict cell-type-specific contact maps, we calculated the similarity between imputed contact maps with HiCRep [13], which is the weighted sum of SCC. In total, our study imputed high-resolution chromatin contact maps for sixteen human cell lines. The pairwise similarity matrix between the sixteen cell lines is visualized in Supplementary Figure 5. As a control, we also calculated the HiCRep scores between experimental Micro-C contact maps from biological replicates of H1 and HFF. The HiCRep scores between the sixteen cell lines (mean=0.77, std=0.04) are comparable with the scores between H1 and HFF (mean=0.74, std=0.03), whereas HiCRep scores between two biological replicates are as high as 0.85 (H1) and 0.87 (HFF). Therefore, CAESAR-imputed contact maps of human cell lines show similar variability as experimental Micro-C contact maps, indicating CAESAR's capability of distinguishing cell types.

Some of the loops and stripes predicted by CAESAR are also cell type-specific. From the imputed high-resolution chromatin contact maps for the sixteen human cell lines, we first called loops and stripes using the loop and stripe callers described in our manuscript, and achieved sixteen sets of loops and stripes. Aggregated peak analysis (APA) was then carried out across the sixteen human cell lines, in which we piled up the contacts in the called loop and stripe regions, and calculated contact enrichment scores for these sixteen sets of loops and stripes in the sixteen sets of contact maps. The APA analysis produced a 16-by-16 matrix with rows corresponding to the sixteen sets of loops and stripes, and columns corresponding to the sixteen contact maps in which we calculated the contact enrichment scores (Supplementary Figures 6a and 6c). Therefore in this 16-by-16 matrix, diagonal elements are cell-type-matched whereas off-diagonal elements are cell-type-mismatched. Under the null hypothesis that these loops and stripes are not cell-type-specific, the enrichment scores from diagonal elements and those from off-diagonal elements should be similarly distributed. It is observed that chromatin contacts in loop/stripe regions called from one cell type are significantly more enriched in the cell-type-matched contact map predicted by CAESAR (Supplementary Figures 6b and 6d). This demonstrates that some fine-scale structures predicted by CAESAR are cell type-specific.

Supplementary Note 11 The genome-wide attribution analysis of stripes and loops

The integrated gradient can be applied to arbitrary regions of the imputed contact map. Here we show, by calculating the attribution of all stripe regions, we can identify sub-types of stripes.

We selected the stripes which were called on both Micro-C and CAESAR-imputed contact maps. Since the stripes were called at 1 kb resolution, we identified the accurate stripe anchors at 200 bp resolution by selecting the row/column with the largest summation on the Micro-C contact map. The stripe regions were defined as 11×500 long rectangles starting from the stripe anchor on the diagonal and stretching in the same direction as the stripes. We calculated the attribution of all stripe regions with integrated gradient. Only the attribution near the anchors (i.e., 100 bins both upstream and downstream)

were preserved (Supplementary Figure 8b), resulting in 6×201 attribution matrices. We observed a significant difference of attributions between stripes spanning to upstream and downstream directions (Supplementary Figure 8d). Therefore, we flipped the attribution results for all stripes which span to the upstream direction. Afterwards, we used PCA to reduce the dimension from 1,206 (i.e., 6×201) to 50, and then *k*-means to cluster the 50-dim vectors. The 50-dim vectors are further transformed into 2-dim with *t*SNE for visualization.

The attribution near all stripe anchors can be clustered into two groups, in which each group has its characteristic patterns in average attribution and epigenomic features. Cluster 1 stripes have higher CTCF attribution, while cluster 2 stripes have higher H3K4me1 attribution. We inferred that cluster 1 stripes are related to chromatin structure maintenance and cluster 1 stripes are related to regulatory activities, which are referred to as "structural stripes" and "regulatory stripes" respectively (Supplementary Figure 8e).

Similar analysis was performed on loops, in which the attribution on loop anchors was clustered (Supplementary Figure 8c). Two groups of loops are observed. Cluster 1 loops have higher CTCF attribution, while cluster 2 loops have higher H3K4me1/me3 attribution. The two clusters are also referred to as "structural loops" and "regulatory loops" (Supplementary Figure 8f).

Although the sub-types still need to be further explored and experimentally validated, this approach provides interpretable insights into our "black box".

Supplementary Note 12 Supporting evidence for stripes' negative attribution to H3K27ac

Although stripes do overlap with active histone modifications such as H3K27ac and H3K4me3 more frequently [12], CAESAR does not attribute stripes positively to H3K27ac. Since CAESAR is data-driven, the supporting evidence can be found from the original epigenomic data. We observed that, among the 1,000 loci with the highest H3K4me3 signal on test set chromosomes, 374 are stripe anchors. By contrast, among the 1,000 loci with the highest H3K27ac signal on the same chromosomes, only 50 are stripe anchors (Supplementary Figure 9). Therefore, CAESAR correlates stripes with H3K4me3 but regards H3K27ac as a feature of "active regions but not stripes" and attributes it negatively.

Supplementary Note 13 Web server implementation

The imputed high-resolution contact maps are shared on a web server (https://nucleome.dcmb.med.umich.edu/), which allows users to easily navigate these fine-scale chromatin structures, and the corresponding explanatory epigenomic features. The back-end of the server uses python *Flask* with *sqlite*. The front-end of the server uses *bootstrap* framework. The web server utilizes multi-threading to allow multiple users to access it at the same time. Our web server processes host data at multiple ports at localhost. We use *Nginx* to perform the reverse proxy that passes internet requests to them. After contact maps are generated, we run *Nucleome Browser* on our web server. Nucleome Browser is an open platform to integratively and interactively browse coordinate-based genome data. Nucleome Browser extends conventional track-based genome browsing to panel-based genome browsing, thus breaks the linear limitation of stacked tracks view mode. Different panel modules host and render different modality data including visualized tracks and reconstructed 3D chromatin structures.

References

[1] J. Dekker, A. S. Belmont, M. Guttman, V. O. Leshyk, J. T. Lis, S. Lomvardas, L. A. Mirny, C. C. O'Shea, P. J. Park, B. Ren, J. C. Ritland Politz, J. Shendure, S. Zhong, and the 4D Nucleome Network. The 4D nucleome project. *Nature*, 549:219–226, 2017.

[2] Cricket A Sloan, Esther T Chan, Jean M Davidson, Venkat S Malladi, J Seth Strattan, Benjamin C Hitz, Idan Gabdank, Aditi K Narayanan, Marcus Ho, Brian T Lee, et al. ENCODE data at the ENCODE portal. *Nucleic Acids Research*, 44(D1):D726–D732, 2016.

[3] Rongbin Zheng, Changxin Wan, Shenglin Mei, Qian Qin, Qiu Wu, Hanfei Sun, Chen-Hao Chen, Myles Brown, Xiaoyan Zhang, Clifford A Meyer, et al. Cistrome Data Browser: expanded datasets and new tools for gene regulatory analysis. *Nucleic Acids Research*, 47(D1):D729–D735, 2019.

[4] Shenglin Mei, Qian Qin, Qiu Wu, Hanfei Sun, Rongbin Zheng, Chongzhi Zang, Muyuan Zhu, Jiaxin Wu, Xiaohui Shi, Len Taing, et al. Cistrome Data Browser: a data portal for ChIP-Seq and chromatin accessibility data in human and mouse. *Nucleic Acids Research*, page gkw983, 2016.

[5] John Lonsdale, Jeffrey Thomas, Mike Salvatore, Rebecca Phillips, Edmund Lo, Saboor Shad, Richard Hasz, Gary Walters, Fernando Garcia, Nancy Young, et al. The genotype-tissue expression (GTEx) project. *Nature Genetics*, 45(6):580–585, 2013.

[6] Charles P Fulco, Mathias Munschauer, Rockwell Anyoha, Glen Munson, Sharon R Grossman, Elizabeth M Perez, Michael Kane, Brian Cleary, Eric S Lander, and Jesse M Engreitz. Systematic mapping of functional enhancer–promoter connections with CRISPR interference. *Science*, 354(6313):769–773, 2016.

[7] W. J. Kent, C. W. Sugnet, T. S. Furey, K. M. Roskin, T. H. Pringle, A. M. Zahler, and D. Haussler. The Human Genome Browser at UCSC. *Genome Research*, 12:996–1006, 2002.

[8] Ashish Vaswani, Noam Shazeer, Niki Parmar, Jakob Uszkoreit, Llion Jones, Aidan N Gomez, Lukasz Kaiser, and Illia Polosukhin. Attention is all you need. *arXiv preprint arXiv:1706.03762*, 2017.

[9] Vinod Nair and Geoffrey E Hinton. Rectified linear units improve restricted boltzmann machines. In *Proceedings of the 27th International Conference on Machine Learning (ICML-10)*, pages 807–814, 2010.

[10] Yan Zhang, Lin An, Jie Xu, Bo Zhang, W Jim Zheng, Ming Hu, Jijun Tang, and Feng Yue. Enhancing Hi-C data resolution with deep convolutional neural network HiCPlus. *Nature Communications*, 9(1):750, 2018.

[11] Shilu Zhang, Deborah Chasman, Sara Knaack, and Sushmita Roy. In silico prediction of high-resolution Hi-C interaction matrices. *Nature Communications*, 10(1):1–18, 2019.

[12] Laura Vian, Aleksandra Pekowska, Suhas SP Rao, Kyong-Rim Kieffer-Kwon, Seolkyoung Jung, Laura Baranello, Su-Chen Huang, Laila El Khattabi, Marei Dose, Nathanael Pruett, et al. The energetics and physiological impact of cohesin extrusion. *Cell*, 173(5):1165–1178, 2018.

[13] T. Yang, F. Zhang, G. G. Yardımcı, F. Song, R. C. Hardison, W. S. Noble, F. Yue, and Q. Li. HiCRep: assessing the reproducibility of Hi-C data using a stratum-adjusted correlation coefficient. *Genome Research*, 27(11):1939–1949, 2017.
