# Supplementary material for: Connecting high-resolution 3D chromatin organization with epigenomics
Source: Nat Commun. 2022 Apr 19;13:2054. doi: 10.1038/s41467-022-29695-6 (PMC9018831; doi:10.1038/s41467-022-29695-6)
Supplement: Supplementary file 3 — Reporting Summary [file 41467_2022_29695_MOESM3_ESM.pdf]

nature research

Corresponding author(s): Jie Liu

Last updated by author(s): Feb 28, 2022

Reporting Summary

Nature Research wishes to improve the reproducibility of the work that we publish. This form provides structure for consistency and transparency in reporting. For further information on Nature Research policies, see our Editorial Policies and the Editorial Policy Checklist.

Statistics

For all statistical analyses, confirm that the following items are present in the figure legend, table legend, main text, or Methods section.

n/a	Confirmed	
☐	☒	The exact sample size (n) for each experimental group/condition, given as a discrete number and unit of measurement
☒	☐	A statement on whether measurements were taken from distinct samples or whether the same sample was measured repeatedly
☐	☒	The statistical test(s) used AND whether they are one- or two-sided<br>*Only common tests should be described solely by name; describe more complex techniques in the Methods section.*
☒	☐	A description of all covariates tested
☒	☐	A description of any assumptions or corrections, such as tests of normality and adjustment for multiple comparisons
☒	☐	A full description of the statistical parameters including central tendency (e.g. means) or other basic estimates (e.g. regression coefficient) AND variation (e.g. standard deviation) or associated estimates of uncertainty (e.g. confidence intervals)
☐	☒	For null hypothesis testing, the test statistic (e.g. F, t, r) with confidence intervals, effect sizes, degrees of freedom and P value noted<br>*Give P values as exact values whenever suitable.*
☒	☐	For Bayesian analysis, information on the choice of priors and Markov chain Monte Carlo settings
☒	☐	For hierarchical and complex designs, identification of the appropriate level for tests and full reporting of outcomes
☒	☐	Estimates of effect sizes (e.g. Cohen's d, Pearson's r), indicating how they were calculated

Our web collection on statistics for biologists contains articles on many of the points above.

Software and code

Policy information about availability of computer code

Data collection	The data were downloaded direct from the sources shared in Supplementary Tables 2 and 3. No extra software was used.
Data analysis	The algorithm and all following analysis was implemented with Python 3.6.8. The code is shared in https://github.com/liu-bioinfo-lab/caesar. The required environment: Python-3.6.8 with numpy-1.17.4, scipy-1.4.1, keras-2.2.4, tensorflow-1.13.1, matplotlib-3.0.2, pyBigWig-0.3.17, seaborn-0.9.0, and pandas-0.23.4

For manuscripts utilizing custom algorithms or software that are central to the research but not yet described in published literature, software must be made available to editors and reviewers. We strongly encourage code deposition in a community repository (e.g. GitHub). See the Nature Research guidelines for submitting code & software for further information.

Data

Policy information about availability of data

All manuscripts must include a data availability statement. This statement should provide the following information, where applicable:

- Accession codes, unique identifiers, or web links for publicly available datasets
- A list of figures that have associated raw data
- A description of any restrictions on data availability

The datasets we used are listed in Supplementary Note 1 and Supplementary Tables 2 and 3 with accession codes and/or links.

Our figures are generated from the computational results of CAESAR. The imputation results and/or the attribution results of the visualized regions are shared in our GitHub repository (https://github.com/liu-bioinfo-lab/caesar). The entire imputed contact maps of tissues/cell lines are shared on our web server (https://nucleome.dcmb.med.umich.edu/). All results can be reproduced by our published code.
All data used in our model training, tuning, and evaluation are publicly available.

Field-specific reporting

Please select the one below that is the best fit for your research. If you are not sure, read the appropriate sections before making your selection.

☒ Life sciences ☐ Behavioural & social sciences ☐ Ecological, evolutionary & environmental sciences

For a reference copy of the document with all sections, see nature.com/documents/nr-reporting-summary-flat.pdf

Life sciences study design

All studies must disclose on these points even when the disclosure is negative.

Sample size	The 23 chromosomes (1-22 + X) are split into train, tune, and test sets to ensure similar total lengths of each set.<br>We used the train set to train the parameters and the tune set to choose hyperparameters.<br>Evaluation experiments in our paper were conducted on the test set only.
Data exclusions	No data were excluded.
Replication	During tuning, we repeat the model training process with different hyperparameter settings.<br>When evaluating the trained model, the results are generated with reproducible code and will not change in a repeating experiment.
Randomization	In our study, train/tune/test sets were chosen to ensure similar total chromosome lengths, and each set has both long and short chromosomes.
Blinding	N/A since no investigators were included in our study.

Reporting for specific materials, systems and methods

We require information from authors about some types of materials, experimental systems and methods used in many studies. Here, indicate whether each material, system or method listed is relevant to your study. If you are not sure if a list item applies to your research, read the appropriate section before selecting a response.

Materials & experimental systems

n/a	Involved in the study	
☒	☐	Antibodies
☒	☐	Eukaryotic cell lines
☒	☐	Palaeontology and archaeology
☒	☐	Animals and other organisms
☒	☐	Human research participants
☒	☐	Clinical data
☒	☐	Dual use research of concern

Methods

n/a	Involved in the study	
☒	☐	ChIP-seq
☒	☐	Flow cytometry
☒	☐	MRI-based neuroimaging
